# Application of Autofluorescence Endoscopy for Colorectal Cancer Screening: Rationale and an Update

**DOI:** 10.1155/2012/971383

**Published:** 2011-12-06

**Authors:** Hiroyuki Aihara, Hisao Tajiri, Takeshi Suzuki

**Affiliations:** ^1^Department of Endoscopy, The Jikei University School of Medicine, 3-25-8 Nishi Shinbashi, Minato-ku, Tokyo 105-8461, Japan; ^2^Division of Gastroenterology and Hepatology, Department of Internal Medicine, The Jikei University School of Medicine, 3-25-8 Nishi Shinbashi, Minato-ku, Tokyo 105-8461, Japan

## Abstract

As the result of basic researches, several intravital fluorophores have been determined so far in human colorectal tissue. Autofluorescence endoscopy (AFE) can detect slight alterations in their distribution and concentration during the colorectal carcinogenesis process and, thus facilitate noninvasive screening colonoscopies without the need for fluorescent substances or staining reagents to be administered. While detecting faint autofluorescence intensity by conventional fiberoptic endoscopy remains challenging, the latest AFE system with high-resolution videoendoscope capabilities enables such detection by using a false-color display algorithm. To this end, the diagnostic benefits of AFE have been reported in several multicenter randomized controlled studies of colorectal cancer (CRC) screening and differential diagnosis. CRC screening using the latest AFE technology could, therefore, lead to future reductions in CRC mortality.

## 1. Introduction

Early detection and removal of colorectal adenomatous polyps is essential in reducing the mortality rate of colorectal cancer (CRC). Although there are several modalities for CRC screening, colonoscopy is considered the most effective procedure, allowing direct visualization and on-site treatment of the encountered lesions. However, minute or flat-type polyps are hard to detect even by conventional colonoscopy [1]. Narrow-band imaging (NBI) has been widely applied for the diagnosis of colorectal neoplasm during colonoscopy [2–4]. However, recent prospective studies [5–7] failed to show the effectiveness of NBI in screening colonoscopies.

Autofluorescence endoscopy (AFE) is now attracting attention for its potential in improving diagnostic yields for CRC. This technology was shown to detect slight alterations in autofluorescence intensity in the colorectal wall during the carcinogenesis process [8, 9].

## 2. Principle of AFE

### 2.1. Fluorophores in Human Colorectal Tissue

When light is focused onto a molecule, part of the light energy is reflected or scattered, and the rest is absorbed. The energy status of the molecule shifts from a ground state to a high-vibration energy state—this is known as excitation. Excess energy is emitted as thermal energy or consumed as vibrational energy when the molecules revert to their ground state. However, naturally fluorescent molecules in tissue release such excess energy as autofluorescence, which can be detected and measured.

Fluorophores determined so far in human colorectal tissue include collagen, which forms the basement membrane and the submucosal layer, NADH and FAD, which exist mainly in gland cell mitochondria and lysosomal granules, and porphyrin in the mitochondria of red blood cells and gland cells.

Autofluorescence emission has been reported mainly with respect to collagen distributed throughout the submucosal colorectal layer [8], with lower autofluorescence intensity also detected in neoplastic tissues. This reduced intensity is attributed to the attenuated optical penetrability, both for the excitation light and the autofluorescence, caused by the increased mucosal thickness and glandular density of neoplasm [9].

### 2.2. Fluorescence Endoscopy

Several studies focusing on fluorescence endoscopy combined with topical application of fluorescent substances such as porphyrin [10], tetracycline [11], or fluorescein [11] from the mid-20th century failed to reveal a diagnostic value. Low tissue specificity and technology deficits resulted in a failure to detect faint fluorescence intensity. However, a recent prospective study by Mayinger et al. [12] on the detection rate of colonic neoplastic lesions by photodynamic diagnosis (PDD) using fluorescence endoscopy (13902 PIKS; KARL STORZ, Tuttlingen, Germany) with topical application of 5-aminolevulinic acid (5-ALA), a precursor of porphyrin, and hexaminolevulinate (HAL), a derivative of 5-ALA, found that both applied fluorophores have an affinity for neoplastic tissues. In their study, PDD detected 28% more neoplastic lesions than white light endoscopy (WLE). Although PDD carries the inherent risk of complications such as photosensitivity, the modality has shown favorable diagnostic yields for detecting dysplasia in patients with ulcerative colitis [13] and Barrett's esophagus [14].

### 2.3. Autofluorescence Endoscopy

AFE detects intravital fluorescent substances without administration of exogenous fluorescent agents. Firstly developed as an autofluorescence bronchoscope (light-induced fluorescence endoscopy: LIFE, Xillix Technologies, British Columbia, Canada) [15], the technology was consequently applied to gastrointestinal endoscopy (LIFE-GI) [16]. This system uses analog equipment based on a fiberoptic endoscope and displays the ratio of green and red autofluorescence intensities as false color. A study in 2001 by Haringsma et al. [17] revealed that AFE based on this technology successfully visualized flat lesions 10 mm or larger in size, which were difficult to detect by WLE. However, this system had practical use problems in a clinical setting, as it was equipped with a heavy camera attached to the endoscope eyepiece [18].

**Figure 1:**
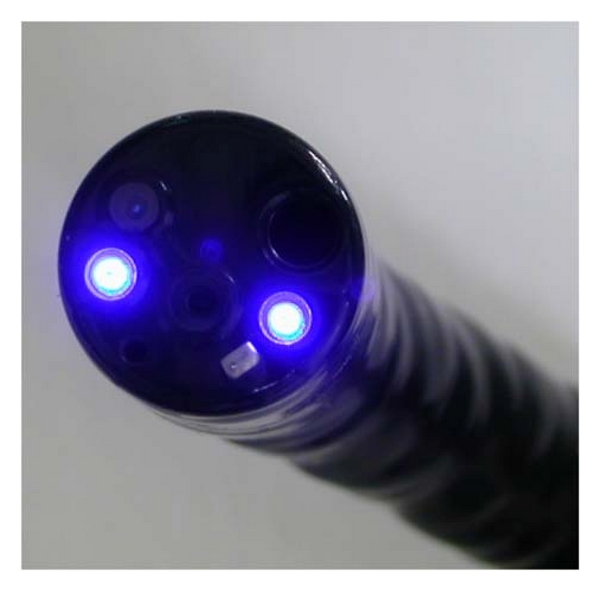
High-resolution videoendoscope (CF-FH260AZI) used in the autofluorescence imaging system (Olympus Medical Systems Corp, Tokyo, Japan).

**Figure 2:**
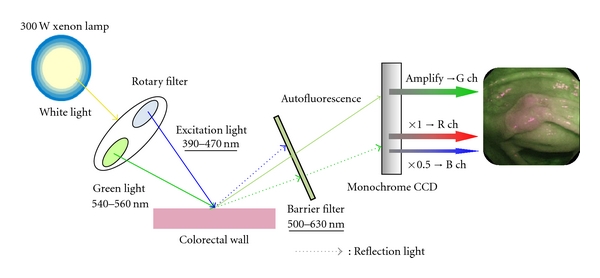
Schematic diagram of the AFI system [19]: white light emitted from a 300-W xenon lamp in the light source is separated with a rotary filter into an excitation light with a wavelength range of 390 to 470 nm and a green light of 540 to 560 nm wavelength. These fractionated lights radiate sequentially during the observation period. A barrier filter to remove reflected excitation light is set in front of a monochrome charge-coupled device. Light of 500 to 630 nm wavelength is selectively detected from both autofluorescence and reflected green light. A false color image is produced by allocating the detected and amplified autofluorescence signal to the green (G) channel and the reflected signal of green light to the red (R) and blue (B) channels in the ratio of 1 to 0.5.

**Figure 3:**
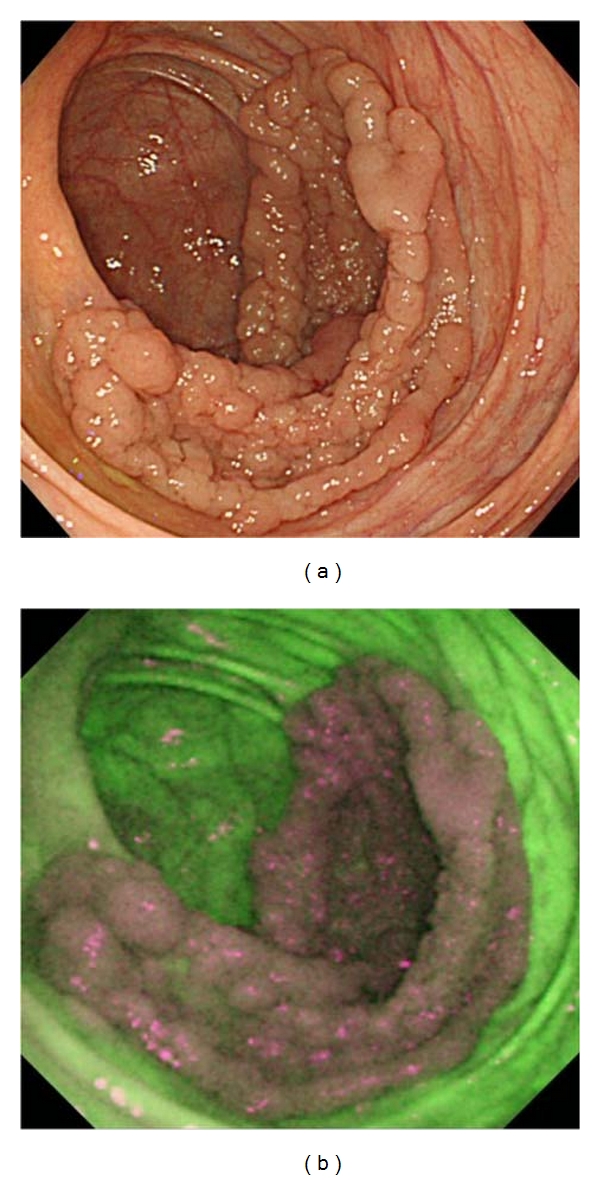
WLE image of 5-cm lateral spreading tumor (granular type) in cecum (a). With AFE, the lesion appears purple, which provides a strong color contrast with the surrounding normal mucosa shown in green (b).

The autofluorescence imaging system from Olympus (AFI system) is the latest AFE system and is equipped with high-resolution videoendoscope capabilities (CF-FH260AZI, Figure 1). This system uses a switching function between the WLE and AFE mode and the NBI mode, a zoom function, and variable stiffness function.  Figure 2 sets out mechanistic details of the AFI system, in which false color images are ultimately produced by allocating the amplified autofluorescence signal to the green (G) channel and the reflected signal of green light to the red (R) and blue (B) channels in the ratio of 1 to 0.5. The endoscopic image is displayed in false color; areas with low and high autofluorescence intensity are shown in purple and green tones, respectively.  Figure 3(a) shows a WLE image of a 5-cm lateral spreading tumor (granular type) in cecum, which is displayed by AFE as purple, thus, providing a strong color contrast with the surrounding normal mucosa shown in green (Figure 3(b)).

A comparative study [20] between the LIFE-GI and AFI systems for differentially diagnosing hyperplastic lesions from colorectal adenomas revealed that sensitivity and specificity were 87% and 71% for LIFE-GI and 89% and 81% for AFI, respectively.

## 3. AFE in CRC Screening

Based on the advantage of AFE that colorectal lesions are displayed in purple, which is like a “red flag” in the surrounding normal colorectal mucosa shown in green, several randomized clinical trials have focused on the diagnostic utility of AFE in screening by colonoscopy. In a randomized controlled study using the AFI system [21], a modified back-to-back colonoscopy using AFE and WLE was conducted for 167 patients in the right-sided colon by a single, experienced colonoscopist. The patients were randomized to undergo the first colonoscopy with either AFE or WLE (group A: AFE-WLE, group B: WLE-AFE). Among all detected polyps, the number of neoplastic lesions detected by AFE and WLE colonoscopy was 92 and 69, respectively. Among 66 neoplastic lesions detected in group A, 47 (71%) were detected at the first AFE. In contrast, among 95 neoplastic lesions in group B, only 50 (53%) were detected at the first WLE, and 45 (47%) lesions were detected by the subsequently performed AFE. This indicated that significantly more neoplastic lesions were missed by WLE compared with AFE (*P* = 0.02).

A back-to-back comparative study by Ramsoekh et al. [22] analyzed the sensitivity of AFE and WLE for the detection of colorectal adenomas in high-risk patients from families with the Lynch syndrome or familial CRC. A total of 75 asymptomatic patients were examined with either WLE followed by AFE or AFE followed by WLE. Back-to-back colonoscopy was performed by two blinded endoscopists. WLE identified adenomas in 28/41 patients and AFE in 37/41 patients, representing a 32% difference in detection efficacy. In total, 95 adenomas were detected, 65 by WLE and 87 by AFE, indicating a significantly higher sensitivity of AFE compared with WLE (92% versus 68%; *P* = 0.001). In addition, the additionally detected adenomas with AFE were significantly smaller than the adenomas detected by WLE (mean 3.0 mm versus 4.9 mm; *P* < 0.01).

Although early detection and removal of colorectal adenomas is considered the most effective way of preventing colorectal cancer progression [19, 23], the impact of these reported higher detection rates of adenomas by AFE on CRC screening is still unclear due to the relatively small study populations tested thus far. Moreover, whether AFE is useful for detecting those depressed colorectal lesions with higher malignant potential is still unclear. These points should be verified in future large-volume multicenter trials.

## 4. AFE in the Differential Diagnosis of Colorectal Neoplasm

The false color range in AFE is determined based on the calculation of balance between autofluorescence intensity and reflected green light intensity (Green/Red, G/R ratio), and this balance could be affected by thickness of the lesion, degree of vascularity, and glandular density. We numerically analyzed the color tone of colorectal lesions in AFE using special color analysis software [24]. A total of 103 colorectal lesions (22 nonneoplastic and 81 neoplastic lesions) were analyzed, and the mean G/R ratio was significantly higher in nonneoplastic lesions (1.17 (95% CI, 1.10–1.24), *n* = 22) than in neoplastic lesions (0.65 (95% CI, 0.63–0.68), *n* = 81) (*P* < 0.001). Under receiver operating characteristic analysis, with a cut-off value of 1.01 for G/R ratio, it was shown that AFE had a sensitivity and specificity of 98.8% and 86.4%, respectively. This result indicated that the color tone in AFE might directly visualize pathological features of colorectal lesions, and its analysis may facilitate the automated optical diagnosis of colorectal neoplastic lesions in the future.

## 5. Limitations of the AFI System

Despite more advantages with the latest AFE technology, the system still has some limitations that need to be overcome for its full potential to be realised. The outside diameters of the distal end and insertion tube are relatively thick (14.8 and 13.2 mm, resp.) compared to those used in conventional colonoscopy. This might limit maneuverability and, thus, hinder polyp detection, especially of those lesions harbored behind folds or flexures. Use of a transparent hood (TH) in AFE was shown to improve detection rates for colorectal neoplasms [25]. In this study, 561 patients were allocated among four groups: WLE alone, WLI without TH; WLI + TH, WLE with TH; AFE alone, AFE without TH; AFI + TH, AFE with TH. The neoplasm detection rate (95% confidence interval) in the AFI + TH group was significantly higher than that in the WLE alone group (1.96 [1.50–2.43] versus 1.19 [0.93–1.44]; *P* = 0.023).

 The AFI system has two other limitations—delayed display and low image resolution. In our system, both video-frame rate and image resolution were reduced to create false-color images employing very faint autofluorescence intensity. In the future these factors should be overcome with system refinements so that CRC screening using this technology becomes more practical.

## 6. Conclusion

In this paper, we reviewed several papers that focused on the diagnostic value of AFE for CRC screening. We anticipate that AFE may contribute to future reductions in CRC mortality.
